# SREBP-1c Deficiency Affects Hippocampal Micromorphometry and Hippocampus-Dependent Memory Ability in Mice

**DOI:** 10.3390/ijms22116103

**Published:** 2021-06-05

**Authors:** Mary Jasmin Ang, Sueun Lee, Mai Wada, Poornima D. E. Weerasinghe-Mudiyanselage, Sung-Ho Kim, Taekyun Shin, Tae-Il Jeon, Seung-Soon Im, Changjong Moon

**Affiliations:** 1 Department of Veterinary Anatomy and Animal Behavior, College of Veterinary Medicine and BK21 FOUR Program, Chonnam National University, Gwangju 61186, Korea; 166371@jnu.ac.kr (M.J.A.); leese@kiom.re.kr (S.L.); wataametokatatumuri@gmail.com (M.W.); 208314@jnu.ac.kr (P.D.E.W.-M.); shokim@chonnam.ac.kr (S.-H.K.); 2 Department of Basic Veterinary Sciences, College of Veterinary Medicine, University of the Philippines Los Baños, Los Baños 4031, Philippines; 3 Herbal Medicine Resources Research Center, Korea Institute of Oriental Medicine, Naju 58245, Korea; 4 Department of Veterinary Anatomy, College of Veterinary Medicine, Jeju National University, Jeju 63243, Korea; shint@jejunu.ac.kr; 5 Department of Animal Science, College of Agriculture and Life Science, Chonnam National University, Gwangju 61186, Korea; tjeon@chonnam.ac.kr; 6 Department of Physiology, Keimyung University School of Medicine, Daegu 42601, Korea; ssim73@kmu.ac.kr

**Keywords:** SREBP-1c, hippocampal neuron, dendritic complexity, structural plasticity, behavioral aberrations

## Abstract

Changes in structural and functional neuroplasticity have been implicated in various neurological disorders. Sterol regulatory element-binding protein (SREBP)-1c is a critical regulatory molecule of lipid homeostasis in the brain. Recently, our findings have shown the potential involvement of SREBP-1c deficiency in the alteration of novel modulatory molecules in the hippocampus and occurrence of schizophrenia-like behaviors in mice. However, the possible underlying mechanisms, related to neuronal plasticity in the hippocampus, are yet to be elucidated. In this study, we investigated the hippocampus-dependent memory function and neuronal architecture of hippocampal neurons in SREBP-1c knockout (KO) mice. During the passive avoidance test, SREBP-1c KO mice showed memory impairment. Based on Golgi staining, the dendritic complexity, length, and branch points were significantly decreased in the apical *cornu ammonis* (CA) 1, CA3, and dentate gyrus (DG) subregions of the hippocampi of SREBP-1c KO mice, compared with those of wild-type (WT) mice. Additionally, significant decreases in the dendritic diameters were detected in the CA3 and DG subregions, and spine density was also significantly decreased in the apical CA3 subregion of the hippocampi of KO mice, compared with that of WT mice. Alterations in the proportions of stubby and thin-shaped dendritic spines were observed in the apical subcompartments of CA1 and CA3 in the hippocampi of KO mice. Furthermore, the corresponding differential decreases in the levels of SREBP-1 expression in the hippocampal subregions (particularly, a significant decrease in the level in the CA3) were detected by immunofluorescence. This study suggests that the contributions of SREBP-1c to the structural plasticity of the mouse hippocampus may have underlain the behavioral alterations. These findings offer insights into the critical role of SREBP-1c in hippocampal functioning in mice.

## 1. Introduction

Alterations in neuronal structure and function have been implicated in the pathophysiology of various neurological disorders [1]. In the brain, the hippocampus is one of the few remaining regions capable of undergoing neuronal plasticity even during adulthood, rendering it especially susceptible to structural and functional alterations [2,3]. These hippocampal alterations further translate to abnormalities of emotions, learning, and memory [4]. These hippocampal abnormalities in volume [5,6], synaptic circuitry [7], and cytoarchitecture [8] have been reported in patients with neurological diseases, including schizophrenia. Rodent models of hippocampal lesions also demonstrate congruent pharmacological, anatomical, and behavioral phenotypes of schizophrenia [9].

Lipids are vital for brain function as they constitute the bulk of the brain and they function in cellular signaling, myelin generation, and neuroplasticity [10]. The brain is largely dependent on de novo lipid synthesis as the blood–brain barrier limits the exogenous lipid transport into the brain [11,12]. Sterol regulatory element-binding proteins (SREBPs) are considered the master regulators of lipid synthesis [13]. SREBPs are transcription factors, controlling both cholestero- and lipogenesis, via the activation of enzymatic cascades required for the synthesis of endogenous cholesterol, fatty acids, and triglycerides [14]. In mammals, three SREBP isoforms have been described, of which SREBP-1a and SREBP-1c are both produced from different transcription sites (exon 1a and exon 1c) of the same *Srebf-1* gene [15], while SREBP-2 originates from the *Srebf-2* gene [16]. SREBP-1c is the predominant isoform expressed in most tissues, including the white adipose tissue, liver, and brain. SREBP-1a is predominantly expressed in cellular proliferative tissues, such as the spleen and intestine [17], while SREBP-2 is ubiquitously expressed throughout the body at amounts approximately equal to the total of the SREBP-1a and 1c expression [18]. Although there is a commonality in their activities, SREBP-1c favors the control of fatty acid and triglyceride synthesis, while SREBP-2 mainly regulates cholesterol synthesis and SREBP-1a does not display any particular preference [13].

Previously, we found that mice lacking SREBP-1c demonstrate schizophrenia-like behavioral symptoms [19]. Furthermore, we found that novel genes (e.g., glucagon-like peptide 2 receptor (*Glp2r*), necdin (*Ndn*), and Erb-B2 receptor tyrosine kinase 4) were significantly altered in the hippocampus of SREBP-1c knockout (KO) mice, compared with those in wild-type (WT) mice [20]. However, the mechanisms possibly underlying these abnormal behaviors and altered molecular expressions need further examination. Thus, we sought to investigate the potential changes in functional and structural neuroplasticity of the hippocampus of SREBP-1c KO mice, which may provide new insights into the role of SREBP-1c in the hippocampus and its potential relevance to the development of animal experimental systems for neurological disorders such as schizophrenia.

In this study, we first confirmed hippocampal function in SREBP-1c KO mice by measuring hippocampus-dependent learning and memory ability using the passive avoidance task. Subsequently, we investigated the structural neuroplasticity of the hippocampi of SREBP-1c KO mice by measuring various parameters of dendritic and spine morphology in the different subregions, including the *cornu ammonis* (CA) 1, CA3, and dentate gyrus (DG); we also characterized the different changes in the morphological parameters in the various subregions. Further, the levels of SREBP-1 expression in the various subregions of mice hippocampi were analyzed. Finally, the possible association between the alteration of structural plasticity and lack of SREBP-1c expression will be discussed.

## 2. Results

### 2.1. SREBP-1c KO Mice Demonstrate Significantly Decreased Hippocampus-Dependent Memory Retention

**Figure 1:**
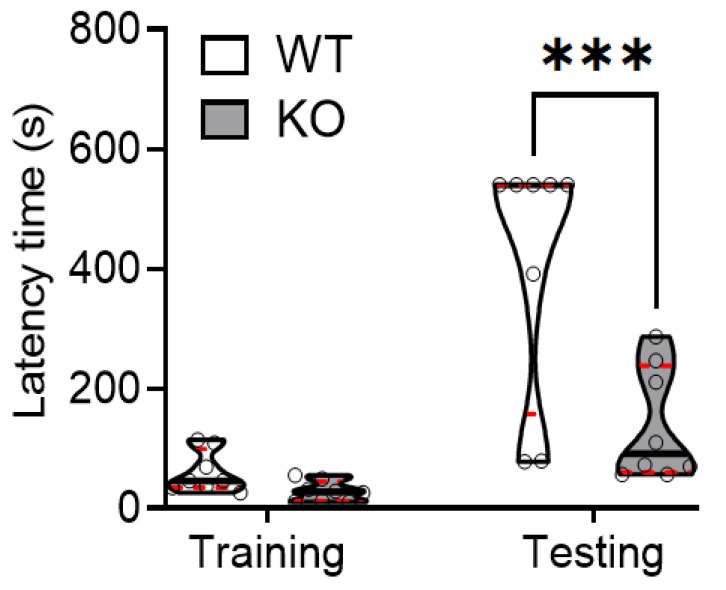
SREBP-1c deficiency significantly impairs learning and memory function in mice. Using the passive avoidance test, WT and SREBP-1c KO mice were trained using mild electrical foot shocks. Both groups were tested 24 h after training. Cross-over latency (s) was assessed to evaluate memory retention. SREBP-1c KO mice showed a significantly lesser increase in the cross-over latency before entering the dark room during testing than WT mice (WT: 406 ± 208.77 s, KO: 138.3 ± 94.40 s, *n* = 8 per group; F_interaction_ (1,14) = 10.17, *p* = 0.0002). The upper and lower dashed red lines signify the upper and lower quartiles, respectively, and the median is represented by a solid black line within a violin plot. *** *p* < 0.001 vs. WT. WT, wild-type littermate; KO, SREBP-1c KO group.

Previously, we observed that SREBP-1c KO mice had altered behaviors suggestive of the positive and negative symptoms of schizophrenia, but not its cognitive symptoms. To further assess cognitive symptoms, we used the passive avoidance task as a hippocampus-dependent paradigm. During the training period, both WT and KO mice had short cross-over latencies before entering the dark compartment where they received a foot shock. Interestingly, during the testing period, memory retention was significantly reduced in KO mice, compared with WT mice. This was evidenced by the significantly shorter increase in the cross-over latency during the testing period (Figure 1).

### 2.2. SREBP-1c KO Mice Display Altered Dendritic Morphology in the Hippocampus

**Figure 2:**
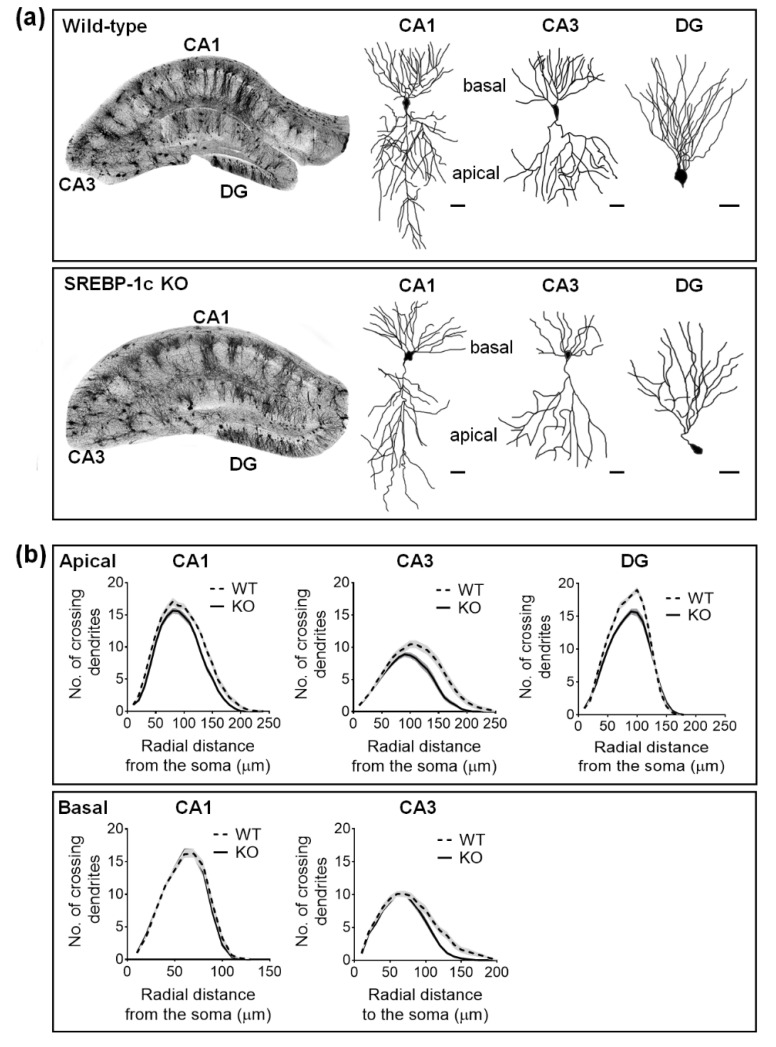
SREBP-1c KO mice display impaired dendritic complexity in the CA1, CA3, and DG subregions of the hippocampus. The figures show the representative images of the Golgi-stained hippocampus and subregional neurons in WT and SREBP-1c KO mice (**a**). The line graphs show the mean number of intersections per 10 µm radial unit distance from the soma (0) for apical ((**b**); upper) and basal ((**b**); lower) dendrites. Data are expressed as means ± standard errors (SEs) of 10 neurons of each subregion in each mouse (*n* = 40 dendrites/group). The scale bars in (**a**) represent 25 µm. CA, *cornu ammonis*; DG, dentate gyrus; WT, wild-type littermate; KO, SREBP-1c KO group.

Sholl analysis was used to quantify the dendritic complexity of the Golgi-stained neurons. The total number of dendritic intersections was recorded per 10 µm incremental radial distance from the soma. Overall, SREBP-1c KO mice, compared with WT mice, had decreased dendritic complexity (Figure 2a). The complexity was significantly reduced in apical CA1, apical and basal CA3, and DG, but not in the basal subcompartment of CA1 (Figure 2b). Apical CA1 dendrites in SREBP-1c KO mice demonstrated significantly fewer intersections than WT mice at the Sholl radii of 130–150 μm from the soma (Figure 2b, upper left line graphs). The number of intersections of the CA3 dendrites in SREBP-1c KO mice, compared with those in WT mice, were significantly reduced in the apical subcompartment at the Sholl radii of 110–180 μm from the soma (Figure 2b, upper middle line graphs) and in the basal subcompartment at the Sholl radii of 110–140 μm from the soma (Figure 2b, lower right line graphs). The DG granule cell dendrites of KO mice also had significantly less intersections than those of WT mice at Sholl radii of 70–110 μm from the soma (Figure 2b, upper right line graphs). However, no significant differences between SREBP-1c KO and WT mice in the basal compartment of the CA1 subregion were observed (Figure 2b, lower left line graphs). Table 1 shows the results of the two-way ANOVA tests for the effects of the genotype and distance on dendritic arborization (mean no. of crossing dendrites) in each hippocampal subregion of WT and KO mice measured by Sholl analysis (*n* = 40 neurons/group).

**Figure 3:**
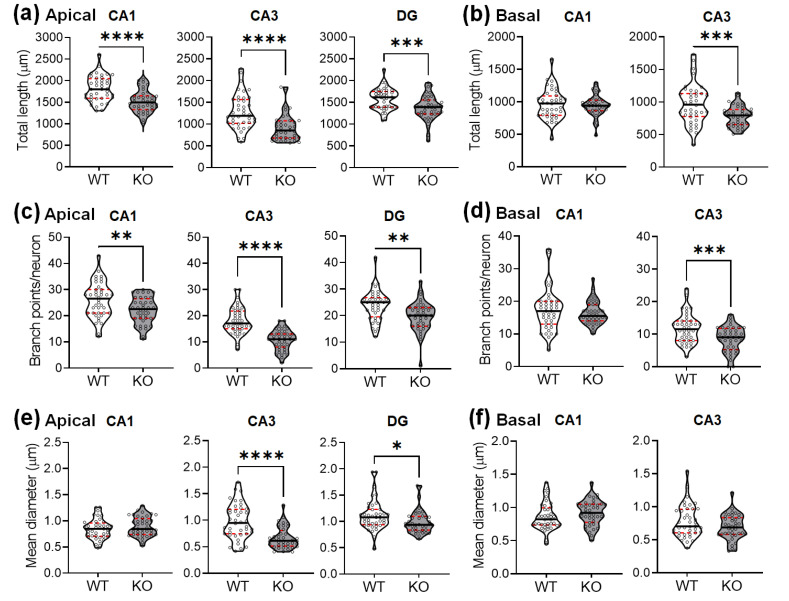
SREBP-1c KO mice show decreases in the length, number of branch points, and diameter of the dendrites in the hippocampus. Violin plots show the total length (**a**,**b**), number of branch points (**c**,**d**), and mean diameter (**e**,**f**) of the dendrites in each subregion (CA1, CA3, and DG). The upper and lower dashed red lines signify the upper and lower quartiles, respectively, and the median is represented by a solid black line within the violin plots. * *p* < 0.05, ** *p* < 0.01, *** *p* < 0.001, and **** *p* < 0.0001 vs. WT (*n* = 40 dendrites/group). CA, *cornu ammonis*; DG, dentate gyrus; WT, wild-type littermate; KO, SREBP-1c KO group.

The total dendritic length was also significantly reduced in the hippocampi of SREBP-1c KO mice, compared with those of WT mice (Figure 3a,b). Deficiencies were observed in the apical CA1 (Figure 3a, left violin plots), apical CA3 (Figure 3a, middle violin plots), DG subregions (Figure 3a, right violin plots), and basal CA3 (Figure 3b, right violin plots), but not in the basal subcompartment of CA1 (Figure 3b, left violin plots).

A corresponding decrease in the dendritic branching points was also observed in the hippocampi of KO mice compared with those of WT mice (Figure 3c,d). The branch points per neuron were reduced in the apical CA1 (Figure 3c, left violin plots), apical CA3 (Figure 3c, middle violin plots), DG subregions (Figure 3c, right violin plots), and basal CA3 (Figure 3d, right violin plots), but not in the basal subcompartment of CA1 (Figure 3d, left violin plots).

The Golgi-stained neurons were also examined for variations in the dendritic thickness in WT and KO mice (Figure 3e,f). The dendritic diameters were significantly reduced in the apical CA3 (Figure 3e, middle violin plots) and DG subregions (Figure 3e, right violin plots) of KO mice, but not in the other areas (CA1 apical: Figure 3e, left violin plots; CA1 basal: Figure 3f, left violin plots; CA3 basal: Figure 3f, right violin plots).

Table 2 shows the results of Student’s *t*-tests for total dendritic lengths, branch points, and thickness of the hippocampal neurons in each subregion between WT and KO mice (*n* = 40 neurons/group).

### 2.3. SREBP-1c KO Mice Show Altered Dendritic Spine Density and Morphology in the Hippocampus

**Figure 4:**
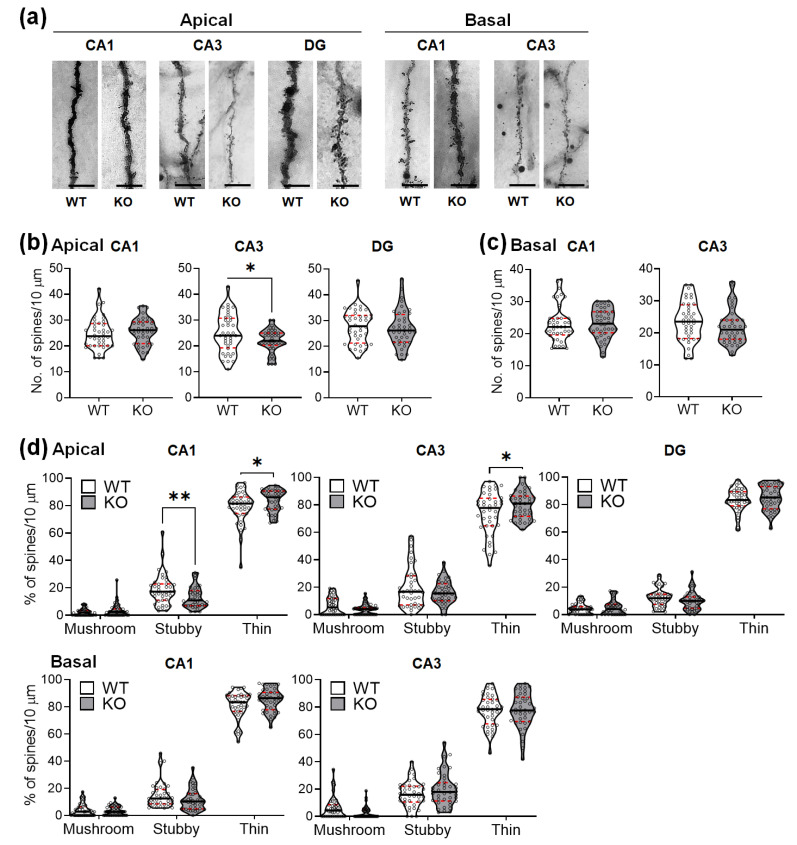
SREBP-1c KO mice show altered dendritic spine density and morphological proportions in the hippocampus. Representative images from each cohort show dendritic segments and their spines along Golgi-impregnated neurons (**a**). Violin plots show the total number of spines in each subregion (CA1, CA3, and DG) (**b**,**c**). Violin plots show the proportions of the spines categorized by morphology in each subregion (CA1, CA3, and DG) (**d**). Upper and lower dashed red lines signify the upper and lower quartiles, respectively, and the median is represented by a solid black line within a violin plot. * *p* < 0.05, ** *p* < 0.01 vs. WT (*n* = 40 dendritic segment/group). The scale bars in the dendritic images represent 5 µm. CA, *cornu ammonis*; DG, dentate gyrus; WT, wild-type littermate; KO, SREBP-1c KO group.

The Golgi-stained neurons were magnified and analyzed for possible changes in the dendritic spines. The mice lacking SREBP-1c had significant alterations in spine density and morphology only in the apical subcompartment of the CA1 and CA3 subregions (Figure 4). The spine densities (number of spines per 10 μm) were not altered in the apical CA1 (Figure 4b, left violin plots), DG (Figure 4b, right violin plots), basal CA1 (Figure 4c, left violin plots), and basal CA3 (Figure 4c, right violin plots), although they significantly decreased in the apical CA3 subregions (Figure 4b, middle violin plots). Table 3 shows the results of Student’s *t*-tests for the dendritic spine density (per dendritic segments of 10 μm) of the hippocampal neurons in each subregion between WT and KO mice (*n* = 40 dendritic segments/group).

Changes in the proportions of different dendritic spine morphologies were observed in the apical CA1 and apical CA3 subregions. Stubby spines were significantly reduced in the apical CA1 subregion (Figure 4d, upper left violin plots), but not in the other areas. In contrast, the thin spines were significantly increased in the apical CA1 (Figure 4d, upper left violin plots) and apical CA3 subregions (Figure 4d, upper middle violin plots), but not in the other subregions. No significant changes in the proportion of mushroom-shaped spines were observed. Table 4 shows the results of two-way ANOVA tests for the effects of the genotype and spine morphology on the proportion of dendritic spines in each hippocampal subregion of WT and KO mice (*n* = 40 dendritic segments (10 μm)/group).

### 2.4. The CA3 Subregion of SREBP-1c KO Mice Shows Significantly Greater Decreases in Dendritic and Spine Morphological Parameters Compared to Those of Other Subregions

**Figure 5:**
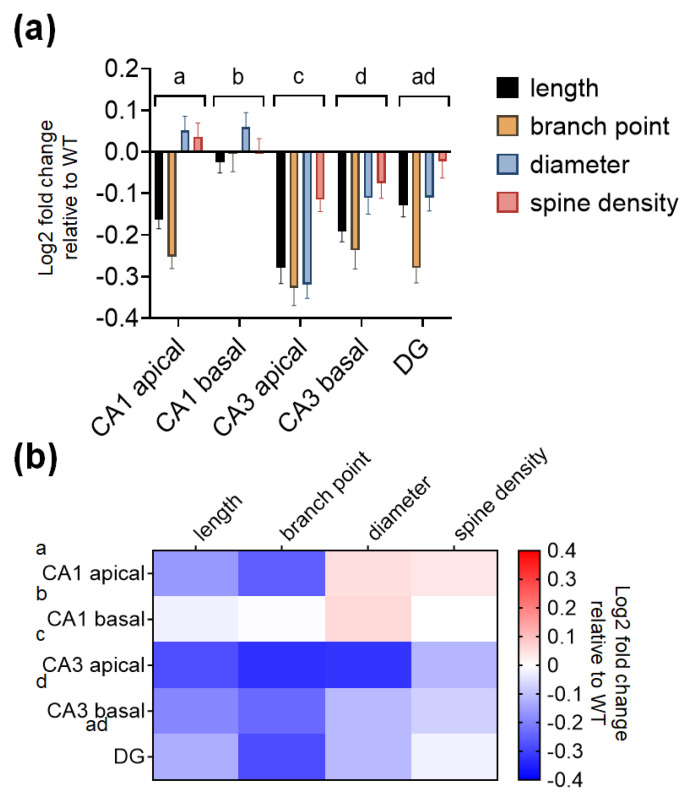
SREBP-1c KO mice show greater morphological alterations in the CA3 than in the other subregions of the hippocampus. Fold changes in dendritic and spine morphological characteristics in WT mice are represented as bar graphs (**a**) and heatmaps (**b**) to better visualize the degree of differences between the different hippocampal subregions (CA1, CA3, and DG). Data are expressed as means ± SEs, and different letters, “a,” “b,” “c,” and “d,” indicate statistically significant differences between the subregions at *p* < 0.05 (*n* = 40 neuron or dendrites/group). Bars with no common letters are significantly different (*p* < 0.05). CA, *cornu ammonis*; DG, dentate gyrus; WT, wild-type littermate group.

To determine if there are subregion-specific differences in the degree of morphological alterations observed, the log2 fold changes in WT mice were computed for each structural parameter. Of the different subregions, the CA3 subregion had the greatest decreases in dendritic length, number of branch points, diameter, and spines compared with the CA1 subregion and DG subregion (Figure 5a, bar graphs). Table 5 shows the results of two-way ANOVA tests for the effects of the hippocampal subregion and morphological parameters on log2 fold changes in KO mice relative to WT mice. Table 6 shows the summary of P-values from Šidăk’s post hoc analyses for the comparison of the different hippocampal subregions in Table 5. To better visualize the subregion-specific differences in the morphological changes, the same set of data is expressed as a heatmap in Figure 5b.

### 2.5. SREBP-1c KO Mice Show Significantly Reduced SREBP-1 Protein Expression in the CA3 Subregion of the Hippocampus

**Figure 6:**
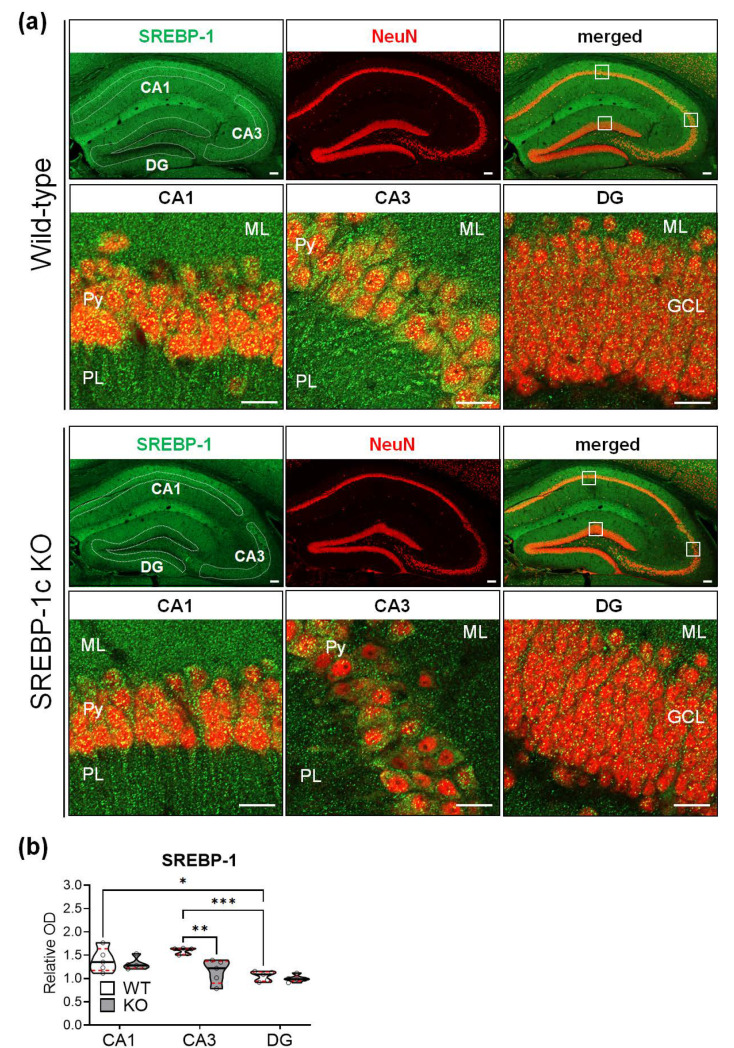
SREBP-1c KO mice show different levels of SREBP-1 protein expressions in each subregion of the hippocampus. Representative immunofluorescence images from each group show expressions of SREBP-1 (green) and NeuN (red)—a neuron-specific marker—in the various hippocampal subregions (CA1, CA3, and DG) (**a**). KO mice showed relatively lesser levels of SREBP-1 expression in all subregions, especially in the CA3 subregion; SREBP-1 was mainly expressed in the pyramidal and granular cell layers of the CA and DG subregions, respectively. Violin plots show the relative intensities of SREBP-1 expression in the pyramidal cell layers (CA1 and CA3) and granular cell layer (DG) in each of the hippocampal subregions (CA1, CA3, and DG) of WT and KO mice (**b**). Upper and lower dashed red lines represent the upper and lower quartiles, respectively, and the median is represented by a solid black line within a violin plot. * *p* < 0.05, ** *p* < 0.01, *** *p* < 0.001 vs. WT (*n* = 5 animals/group). The scale bars represent 100 and 20 µm in the hippocampal and subregion images, respectively. CA, *cornu ammonis*; DG, dentate gyrus; GCL, granular cell layer; ML, molecular layer; PL, polymorphic cell layer; Py, pyramidal cell layer; SREBP, sterol regulatory element-binding protein; NeuN, neuronal nuclei; WT, wild-type littermate; KO, SREBP-1c KO group.

To evaluate the protein expression of SREBP-1 in the hippocampus, the sections were subjected to immunofluorescence staining, and the relative immunoreactive intensities were analyzed using ImageJ software (National Institutes of Health, Bethesda, MD, USA). The SREBP-1 protein was differentially expressed in the subregions of the hippocampi of WT and KO mice; the expressions were most intense in CA3, followed by CA1 and DG (Figure 6a,b). The relative intensities of SREBP-1 expression in the CA1 and DG subregions did not substantially differ in KO and WT mice. However, a significant decrease in SREBP-1 expression was detected in the CA3 subregion compared with that of WT mice of KO mice (Figure 6b, violin plots). Table 7 shows the results of two-way ANOVA tests for the effects of the genotype and hippocampal subregion on the relative immunoreactivity for SREBP-1 in WT and KO mice (*n* = 5 hippocampi/group).

## 3. Discussion

We previously reported schizophrenia-like behavior and altered hippocampal gene expressions in SREBP-1c-deficient mice. In the present study, we expand on these findings by reporting congruent alterations in hippocampal micromorphometry and the function of SREBP-1c-deficient mice, compared with WT mice. Hippocampal function was measured using the passive avoidance task, while neuronal dendritic micromorphometry was analyzed in the tracings of Golgi-impregnated hippocampal neurons.

First, we identified a general impairment in the hippocampal function of SREBP-1c KO mice. We confirmed the deficiency in functional neuroplasticity related to poor learning and memory retention in KO mice using the passive avoidance task. This finding adds to the outcomes of our previous study, which found abnormal behavioral phenotypes resembling positive (hyperlocomotion) and negative (depression, aggression, and social deficit) schizophrenia-like behaviors [19]. Here, we demonstrated the cognitive aspect of schizophrenia-like behavior in SREBP-1c KO mice, which is reported to occur in 80% of the clinical cases and considered a main symptom of schizophrenia [21].

Proper dendritic morphology is essential for normal brain networking and functions, and morphological changes have been implicated in the development and symptoms of various psychiatric disorders, including schizophrenia [22]. In patients with schizophrenia, reductions in dendritic field size, dendritic length, and complexity in cortical or prefrontal cortical neurons have been demonstrated [7,23]. Several crucial molecules, including guanine deaminase cypin [24], Rho GTPases [25], and glutamate receptor-interacting protein [26], have been suggested to be involved in the mechanisms underlying dendritic branching; however, their specific associations with psychiatric disorders have not been established. In the current study, we observed the impairment of dendritic morphology related to decreased dendritic complexity, length, branch point, and diameter in KO mice. SREBPs are important regulators of lipid metabolism in the central nervous system, and SREBP-1c plays a key role in the production of unsaturated fatty acids, especially in the glia [27]. SREBP-1c and the produced fatty acids in the glia appear to play important roles in myelination, neuronal development, neurite outgrowth, synaptogenesis, and synaptic transmission via glia–neuron interactions [27,28,29]. Thus, we suggest that the deficiency in SREBP-1c in the current mouse model may have hindered the growth of dendrites in the hippocampus due to deficiencies in fatty-acid production, although the direct connection between SREPB-1c and the morphological alteration is elusive in this study. Further studies are needed to confirm any possible decreases in the neuronal/glial fatty acid content in the hippocampi of SREBP-1c KO mice.

Additionally, we report the alterations in dendritic spine density and proportions of the different spine subtypes (mushroom, stubby, and thin) in KO mice. Although less prevalent than the impairments of the entire dendrite morphology, the dendritic spine densities were significantly reduced in the apical CA3 subregion of the hippocampi of KO mice. Moreover, the differences in the alterations in the proportions of the spine subtypes were observed in the apical CA1 and CA3 subregions of the KO hippocampi. Dendritic spines constitute the unitary postsynaptic compartment of excitatory connection, and changes in the density and morphology of spines reflect alterations in synaptic strength and connectivity, which in turn affect brain functions [30]. In various psychiatric disorders, including autism spectrum disorders, Alzheimer’s disease, and schizophrenia, spiny synapses are implicated as important substrates of pathoetiology [31]. The alterations of spine density and morphology in disease-specific brain regions may contribute to abnormalities in selected neural circuits, which in turn may underlie the behavioral deficits of the disorders [31]. In schizophrenia, several clinical studies have reported the reduced spine density in some brain regions, including the dorsolateral prefrontal cortex, superior temporal gyrus, and hippocampal CA3 subregions [32,33,34]. The shapes of the spines reflect the efficacy and maturity of synapses. In the present study, the decrease in stubby-shaped spines and increase in thin-shaped spines in the apical CA1 and/or CA3 subregions of KO mice, which reflect a decrease in the volume of the spine head, may influence the decrease in the postsynaptic density and changes in excitatory neurotransmission [35]. Furthermore, the increase in thin spines reflects a greater number of immature synapses [36,37]. The suppression of all potential SREBP activities in vivo via the deletion of the SREBP cleavage-activating protein (SCAP)—a post-translational activator of SREBP—in mice increases the number of thin-shaped spines in the CA1 subregion of the hippocampus [38]. In the current study, the impairments of synaptic structures previously elicited by the broader inactivation of all the SREBP isoforms in SCAP-inactivated mice were retained even with the more specific deletion of SREBP-1c. This suggests the vital role of the SREBP-1c isoform in the maintenance of hippocampal postsynaptic sites and preservation of the population of mature spines. Further studies involving hippocampal synaptosomes from WT and KO mice are warranted to confirm and to elucidate the molecular mechanisms underlying these findings.

Another notable finding in this study is the regional variability of the observed morphological changes. Among the subregions analyzed, CA3 registered the highest degree of changes in the morphological parameters examined. Several clinical and preclinical studies of schizophrenia have also reported morphological alterations of the hippocampal CA3 subregion. Kolomeets et al. [34,39] reported alterations in arborization and a decrease in spine densities, the proportion of mature synapses, and the density of mossy fiber synapses in the CA3 of patients with schizophrenia. In rats prenatally injected with ketamine (a non-competitive inhibitor of glutamate N-methyl-D-aspartate receptors), as animal models of schizophrenia, a significant decrease in the CA3 pyramidal cell layer thickness was reported without changes in cell density [40]. Additionally, the above clinical and animal studies suggest the correlation between the vulnerability of CA3 subregions and behavioral symptoms of schizophrenia. In this study, SREBP-1 expression exhibited the highest degree of reduction in the CA3 subregion of the SREBP-1c KO hippocampi, suggesting that the SREBP-1c isoform, although present in all subregions, was most affected in the CA3 subregion. This may explain the predisposition of the CA3 region, compared with other subregions, to higher degrees of SREBP-1c-related morphological alterations. Congruent structural modifications, although present to a lesser degree, in the CA1 and DG regions might be explained by the intra-hippocampal connectivity between the DG, CA3, and CA1 subregions both anatomically [41] and synaptically (circuitry) [42]. Therefore, any alterations in the signaling of one part might have an overreaching effect on the nearby subregions. Moreover, the remaining SREBP-1 expression present in the hippocampi of SREBP-1c-specific KO mice indirectly represents the remaining proportion of the SREBP-1a isoform in the different subregions. Furthermore, the *Srebf-1a* mRNA expression significantly increased in the whole hippocampal lysates of SREBP-1c KO mice [20]. Therefore, SREBP-1a might have played a role in mitigating the SREBP-1c-induced structural alterations by providing an alternative, compensatory pathway for the de novo lipid synthesis, as it shares common functions with the SREBP-1c isoform. Therefore, additional research investigating the absence of SREBP-1a or both SREBP-1 isoforms would further elucidate the findings of the current study. Experiments confirming SREBP-1c isoform-specific expressions among the hippocampal subregions and mechanistic pathways involved in the morphological alterations observed are warranted. Nevertheless, the importance of the CA3 subregion in both clinical and animal models of schizophrenia—although this varies with specific underlying mechanisms—still provides a sufficient basis for its pronounced susceptibility to alterations in both structural and functional neuroplasticity in the current SREBP-1c KO mice. 

The differentially expressed genes and proteins, suggested as novel molecular candidates related to the hippocampal dysfunction of SREBP-1c KO mice in our previous study, may also provide insights into the neuroplastic alterations found in the current study [20]. *Glp2r*, a gene associated with neuroprotection and neurogenesis [43], and *Ndn*, a gene associated with neuronal differentiation and survival [44,45] and axonal growth [45], decrease especially in the CA3 pyramidal cell layers of SREBP-1c KO mice [20]. Although the proteins have no known direct physical or functional interactions with SREBP-1 based on a protein–protein interaction network analysis [20], their possible indirect interactions with SREBP-1c and/or other neuroplasticity-related molecules cannot be discounted. Further in-depth analyses involving gene–gene and gene–environment interactions are needed to further demonstrate the extent of signaling pathway involvement.

The observations found in the current study are limited to the hippocampi of 3–4-month-old male mice, without any preference for the right or left hippocampus. Some studies have reported sexual dimorphism in the structural [46,47,48] and neurocognitive [48] alterations found in schizophrenia. Additionally, some studies have reported differences between the structures [49,50] and molecular expressions [51,52] of the left and right hippocampi of patients with schizophrenia. Age-dependent differences in structural changes [53,54,55] have also been reported in schizophrenia. To ascertain the practicability of using SREBP-1c KO mice as models of schizophrenia or any other neuropsychiatric diseases, the possible sexual dimorphism, asymmetries between the left and right hippocampi, and age dependence should be further investigated.

## 4. Materials and Methods

### 4.1. Animals and Treatment

Three- to four-month-old SREBP-1c KO mice—generous gifts from Dr. Timothy F. Osborne (Sanford Burnham Prebys Medical Discovery Institute, Orlando, FL, USA)—were maintained in a C57BL/6J strain background as previously described [19,20]. The animals were housed in standard cages in a specific pathogen-free facility maintained on a 12:12 h light/dark cycle at 25 °C. Food and water were provided ad libitum. All experimental and animal handling methods were performed in compliance with the recommendations of the Chonnam National University (6 January 2017; CNU IACUC-YB-2017-01) and the NIH Guide for the Care and Use of Laboratory Animals [56]. All efforts were made to ensure that the number of animals and potential suffering were minimized.

### 4.2. Passive Avoidance

The passive avoidance task employed in this study was performed as outlined in our previous studies [57]. Using a passive avoidance apparatus (UgoBasile, Gemonio, Italy), the test involved training and testing phases that were 24 h apart. During the training, the mice were allowed to explore the lit chamber for 20 s before the trap door was opened, and they were given a foot shock (0.5 mA for 2 s) upon their entry to the dark chamber. The mice were kept in the dark chamber for 20 s before returning them to their home cage. For the testing phase, the mice were placed in the lit chamber again, and the trap door was open after 1 s. The time spent in the lit chamber before entering the dark chamber was recorded as the cross-over latency (s). A limit of 540 s was set for all mice. Shorter latency rates indicate low retention.

### 4.3. Golgi Staining

Golgi staining was used to visualize the dendritic branching complexity and spines of the neurons in the mice hippocampi. The methods and protocols employed were previously described [30,58]. The FD Rapid Golgistain™ Kit (FD Neurotechnologies, Ellicott City, MD, USA) was used, according to the manufacturer’s instructions. Briefly, the brains were placed in a Golgi–Cox solution for 14 days before saturation with sucrose solution for 3–7 days at room temperature (RT). The 200 μm-thick hippocampal sections were prepared on gelatin-coated slides and left to air dry away from light for 1–3 days before processing for Golgi impregnation.

### 4.4. Sholl Analysis

The methods for quantifying dendritic complexity, dendritic length, and the number of branch points were described for our previous studies [30,58] with some modifications. Briefly, Sholl’s concentric method [59] used with the ImageJ (National Institutes of Health, Bethesda, MD, USA) program was used to analyze the neuronal tracings visualized by the 200× Leica DM750 optical microscope (Leica Microsystems, Wetzlar, Germany) and traced using the Leica Application Suite (version 4.12, Leica Microsystems) and Adobe Photoshop CS6 (Adobe Systems, San Jose, CA, USA). Ten neurons per hippocampal subregion were selected from each animal (*n* = 4/group) using the criteria by Morley et al. [60]: (1) the cell body was in the subregion of interest, (2) the staining of the branches was efficient and complete throughout the length, and (3) the branches were isolated from their neighbors. The concentric circles were laid over the neuron starting from the cell body until the end of the longest dendrite at 10 µm intervals, and the number of dendrites intersecting at each circle was analyzed to determine the complexity, length, and branch points.

### 4.5. Analysis of Dendritic Diameter

The dendritic diameters were measured using the ObjectJ plug-in (https://sils.fnwi.uva.nl/bcb/objectj; accessed on 29 December 2020) for ImageJ/Fiji [61] software. The dendritic segments were included in the analysis if they were intact, properly stained, and unbranched. Photomicrographs were taken using a light microscope, the Leica DM750 optical microscope (Leica Microsystems), under 1000× magnification. Using ObjectJ, 8 evenly spaced diameter measurements were taken from 10 µm-long dendritic segments, and 10 segments were analyzed from each animal (*n* = 4/group). Data are expressed as the mean diameter of 8 readings per 10 μm dendritic segments.

### 4.6. Analysis of Dendritic Spine Density and Morphology

The measurement of dendritic spine density and morphology was performed according to our previous studies [20,58]. Briefly, all protruding dendritic spines were counted on 30 µm dendritic segments. As in Section 4.4, only intact, properly stained, and unbranched dendritic segments were included in the analyses under 1000× magnification. The spines were further classified into different morphological subtypes based on criteria by Chakraborti et al. [62]: (1) thin: spines with distinct small heads and elongated necks; (2) mushroom: spines with large heads and distinct necks; and (3) stubby: very short spines with overall stubby appearances and no distinguishable necks. Ten segments were counted from each animal (*n* = 4/group), and spine density was computed as the number of spines per 10 µm of dendritic length.

### 4.7. Immunofluorescence of Free-Floating Sections

The immunofluorescence protocol was based on our previous study with some modifications [58]. The brains were collected and perfused with 4% (*w*/*v*) paraformaldehyde (Sigma-Aldrich, St. Louis, MO, USA) and transferred into 30% (*w*/*v*) sucrose solution for 4 days, and 30 µm-thick coronal sections were made using a frozen sliding microtome (SM2010R; Leica Microsystems) at around −2.06 mm relative to the bregma. Endogenous hydrogen peroxidase activity was neutralized through incubation of the sections in 0.3% (*v*/*v*) hydrogen peroxide in distilled water for 20 min. The sections were blocked with 5% (*v*/*v*) normal goat serum (Vector ABC Elite Kit; Vector Laboratories, Burlingame, CA, USA) plus 1% bovine serum albumin (Sigma-Aldrich) in phosphate-buffered saline (pH 7.4) with 0.3% (*v*/*v*) Triton X-100 for 1 h at RT. The sections were incubated with primary antibodies, rabbit anti-SREBP-1 (1:100; Abcam, Cambridge, UK) and mouse anti-neuronal nuclei (NeuN; 1:100; Merck Millipore, Billerica, MA, USA), diluted in antibody dilution buffer (Invitrogen, Carlsbad, CA, USA) overnight at 4 °C. After thorough washing, the sections were incubated in secondary antibodies, goat anti-rabbit IgG (1:500; Alexa-Fluor 488; Invitrogen) or goat anti-mouse IgG (1:500; Alexa-Fluor 594; Invitrogen), for 1 h at RT. The nuclei were counterstained with 4′,6-diamidino-2-phenylindole (1:200; Thermo Fisher Scientific, Waltham, MA, USA) for 30 min at RT. The immunoreactivities of SREBP-1 in the CA1, CA3, and DG subregions of the hippocampus were analyzed with ImageJ/Fiji software [61] (*n* = 5 mice/group).

### 4.8. Analysis of Differences in the Degree of Morphological Alterations in Each Hippocampal Subregion

The values of log2 fold changes in the total length, number of branch points, diameter, and spine densities of the dendrites in each KO hippocampal subregion relative to the WT subregion were obtained and statistically analyzed using Prism (GraphPad Software, San Diego, CA, USA).

### 4.9. Statistical Analyses

All statistical analyses were performed using Prism (GraphPad Software, San Diego, CA, USA). To identify the differences between the cross-over latencies during the passive avoidance tests and the dendritic complexities of the WT and KO groups and the log2 fold changes in the morphological parameters among subregions, two-way repeated-measures ANOVA was performed followed by multiple comparison tests corrected with Šidăk’s post hoc test for 8 animals and 40 neurons per group, respectively. Unpaired Student’s *t*-tests were used for all other experiments to compare the means of the WT and KO groups. The normality of the data distribution was analyzed using the Shapiro–Wilk test. For all the statistical tests, a p-value of less than 0.05 was considered statistically significant. All the exact p-values are reported in the Results section. Power analysis to determine the minimum required sample size per group was performed at an alpha level of 0.05 to achieve a power of at least 90% using La Morte’s power calculator [63]. The minimum required sample sizes obtained using this method were as follows: 8 mice for the passive avoidance test; 40 neurons for dendritic complexity, dendritic length, dendritic branch points, dendritic diameter, spine density, and spine morphology experiments; and 5 mice for the relative staining intensity values obtained from immunofluorescence experiments. The exact sample sizes used per experiment are reported in the Results section, figures, and figure legends.

## 5. Conclusions

In summary, the present study assessed the impairment of neuroplasticity in the hippocampus of SREBP-1c KO mice, with emphasis on structural changes. Independent of the specific underlying mechanisms yet to be ascertained, the evidence of the possible interplay between the CA3 region-specific (1) increase in morphological alterations and impairments, (2) decrease in SREBP-1 expression, and (3) schizophrenia-like behavior-related mechanisms is presented. Thus, the potential usefulness of SREBP-1c KO mice as animal models of schizophrenia has been emphasized. Furthermore, this study improves our understanding of the critical role of SREBP-1c in maintaining the normal architecture and functioning of the mouse hippocampus.

## Figures and Tables

**Table 1:** Results of two-way ANOVA tests for the effects of the genotype and distance on dendritic arborization (mean no. of crossing dendrites) in each hippocampal subregion of WT and KO mice measured by Sholl analysis (*n* = 40 neurons/group).

	Sholl Radii Distance (μm) ^1^	Šidăk’s Post Hoc Test per Sholl Radii Distance	Two-Way ANOVA
CA1 apical	130, 140, 150	*p* = 0.0001, *p* = 0.0007, *p* = 0.0121	F_interaction_ (23, 1794) = 1.741, *p* = 0.0161
basal	n.s.	n.s.	F_interaction_ (12, 936) = 0.8516, *p* = 0.5969
CA3 apical	110, 120, 130, 140, 150, 160, 170, 180	*p* = 0.0371, *p* = 0.0046, *p* = 0.0004, *p* < 0.0001, *p* < 0.0001, *p* < 0.0001, *p* = 0.0004, *p* = 0.0257	F_interaction_ (24,1872) = 3.985, *p* < 0.0001
basal	110, 120, 130, 140	*p* = 0.0243, *p* = 0.0023, *p* = 0.0002, *p* = 0.0490	F_interaction_ (19,1482) = 2.098, *p* = 0.0037
DG	70, 80, 90, 100, 110	*p* = 0.0014, *p* = 0.0149, *p* = 0.0189, *p* < 0.0001, *p* = 0.0025	F_interaction_ (17, 1326) = 4.031, *p* < 0.0001

^1^ At different radial distances from the neuronal soma, the number of dendritic intersections was quantified in each hippocampal subregion, using Sholl analysis. Radial distances with significantly reduced mean numbers of crossing dendrites between WT and KO. Abbreviations: ANOVA, analysis of variance; CA1, *cornu ammonis* 1; DG, dentate gyrus; KO, SREBP-1c knockout; WT, wild-type; n.s., not significant.

**Table 2:** Results of Student’s *t*-tests for total dendritic lengths, branch points, and thickness of the hippocampal neurons in each subregion between WT and KO mice (*n* = 40 neurons/group).

	WT	KO	Student’s *t*-Test
Total dendritic length (μm)			
CA1 apical	1811.3 ± 296.7	1516 ± 251.8	t(78) = 4.794, *p* < 0.0001
basal	964.3 ± 229.5	940.8 ± 160.4	t(78) = 0.531, *p* = 0.5971
CA3 apical	1281 ± 398	924 ± 308.2	t(78) = 4.482, *p* < 0.0001
basal	967.5 ± 301.2	783 ± 156.1	t(78) = 3.440, *p* = 0.0009
DG	1576 ± 245.1	1374 ± 278.5	t(78) = 3.448, *p* = 0.0009
Dendritic branch points			
CA1 apical	25.9 ± 6.7	22.0 ± 5.0	t(78) = 2.956, *p* = 0.0041
basal	17.3 ± 6.3	16.4 ± 3.6	t(78) = 0.719, *p* = 0.4741
CA3 apical	18.1 ± 5.1	10.6 ± 3.9	t(78) = 7.201, *p* < 0.0001
basal	11.8 ± 4.5	8.5 ± 3.9	t(78) = 3.469, *p* = 0.0009
DG	28.3 ± 5.7	19.7 ± 5.5	t(78) = 3.282, *p* = 0.0015
Dendritic thickness (μm)			
CA1 apical	0.845 ± 0.190	0.890 ± 0.185	t(78) = 1.031, *p* = 0.3056
basal	0.864 ± 0.206	0.916 ± 0.189	t(78) = 1.168, *p* = 0.2462
CA3 apical	0.976 ± 0.325	0.664 ± 0.202	t(78) = 5.162, *p* < 0.0001
basal	0.775 ± 0.244	0.689 ± 0.190	t(78) = 1.760, *p* = 0.0823
DG	1.117 ± 0.291	0.994 ± 0.228	t(78) = 2.013, *p* = 0.0387

The data are expressed as the means ± standard deviation. Abbreviations: CA, *cornu* *ammonis*; DG, dentate gyrus; KO, SREBP-1c knockout; WT, wild-type.

**Table 3:** Results of Student’s *t*-tests for the dendritic spine density (per dendritic segments of 10 μm) of the hippocampal neurons in each subregion between WT and KO mice (*n* = 40 dendritic segments/group).

	WT	KO	Student’s *t*-Test
CA1 apical	24.8 ± 6.0	25.7 ± 5.1	t(78) = 0.7302, *p* = 0.4675
basal	23.1 ± 5.7	23.2 ± 4.5	t(78) = 0.0146, *p* = 0.9884
CA3 apical	24.9 ± 7.5	22.0 ± 4.4	t(78) = 2.108, *p* = 0.0382
basal	23.6 ± 6.2	21.9 ± 5.5	t(78) = 1.362, *p* = 0.1770
DG	27.7 ± 6.5	27.1 ± 7.1	t(78) = 0.4203, *p* = 0.6754

The data are expressed as the means ± standard deviation. Abbreviations: CA, *cornu ammonis*; DG, dentate gyrus; KO, SREBP-1c knockout; WT, wild-type.

**Table 4:** Results of two-way ANOVA tests for the effects of genotype and spine morphology on the proportion of dendritic spines in each hippocampal subregion of WT and KO mice (*n* = 40 dendritic segments (10 μm)/group).

	Mushroom Spines (%)	Stubby Spines (%)	Thin Spines (%)	Two-Way ANOVA
CA1 apical				
WT	2.2 ± 2.6	18.7 ± 11.2	79.1 ± 11.8	F_interaction_ (2, 156) = 5.126, *p* = 0.0070
KO	3.4 ± 4.9	13.0 ± 7.8	83.6 ± 7.9	
	*p* = 0.9026 ^1^	*p* = 0.0082 ^1^	*p* = 0.0487 ^1^	
CA1 basal				
WT	4.0 ± 4.7	14.9 ± 9.3	81.0 ± 10	F_interaction_ (2, 156) = 3.003, *p* = 0.0525
KO	3.3 ± 3.2	11.7 ± 7.9	84.9 ± 8.2	
	*p* = 0.9630 ^1^	*p* = 0.1753 ^1^	*p* = 0.0647 ^1^	
CA3 apical				
WT	6.4 ± 6.3	19.7 ± 14.8	73.8 ± 15.8	F_interaction_ (2, 156) = 3.231, *p* = 0.0422
KO	3.9 ± 3.9	16.2 ± 8.4	79.9 ± 9.8	
	*p* = 0.6465 ^1^	*p* = 0.3666 ^1^	*p* = 0.0357 ^1^	
CA3 basal				
WT	6.3 ± 7.8	16.3 ± 9.4	77.4 ± 11.5	F_interaction_ (2, 156) = 1.599, *p* = 0.2054
KO	2.7 ± 4.3	19.7 ± 12	77.6 ± 12.8	
	*p* = 0.3040 ^1^	*p* = 0.3488 ^1^	*p* = 0.9997 ^1^	
DG				
WT	4.2 ± 3.9	12.3 ± 6.7	83.4 ± 8.3	F_interaction_ (2, 156) = 1.224, *p* = 0.2970
KO	5.1 ± 5.2	9.9 ± 6.8	84.9 ± 8.7	
	*p* = 0.9285 ^1^	*p* = 0.3283 ^1^	*p* = 0.6861 ^1^	

^1^ Šidăk’s post hoc test. The data are expressed as the means ± standard deviation. Abbreviations: ANOVA, analysis of variance; CA, *cornu ammonis*; DG, dentate gyrus; KO, SREBP-1c knockout; WT, wild-type.

**Table 5:** Results of two-way ANOVA tests for the effects of the hippocampal subregion and morphological parameters on log2 fold changes in KO mice relative to WT mice.

	Dendritic Length	Dendritic Branch Point	Dendritic Diameter	Spine Density	Two-Way ANOVA
CA1 apical	−0.16 ± 0.022	−0.25 ± 0.029	0.051 ± 0.035	0.037 ± 0.032	Fi_nteraction_ (12, 780) = 3.718, *p* < 0.0001
basal	−0.02 ± 0.026	−0.004 ± 0.043	0.06 ± 0.035	0.0007 ± 0.031	
CA3 apical	−0.28 ± 0.038	−0.33 ± 0.042	−0.32 ± 0.033	−0.12 ± 0.028	
basal	−0.19 ± 0.026	−0.24 ± 0.045	−0.11 ± 0.039	−0.08 ± 0.037	
DG	−0.13 ± 0.028	−0.28 ± 0.035	−0.11 ± 0.032	−0.02 ± 0.04	

The data are expressed as the means ± standard deviation. Abbreviations: ANOVA, analysis of variance; CA, *cornu ammonis*; DG, dentate gyrus; KO, SREBP-1c knockout; WT, wild-type.

**Table 6:** Summary of the P-values from Šidăk’s post hoc analyses for the comparison of the different hippocampal subregions in Table 5.

	CA1 Apical	CA1 Basal	CA3 Apical	CA3 Basal	DG
CA1 apical		0.0024	<0.0001	0.0330	0.2481
CA1 basal			<0.0001	<0.0001	<0.0001
CA3 apical				0.0001	<0.0001
CA3 basal					0.9978
DG					

Abbreviations: CA, cornu ammonis; DG, dentate gyrus.

**Table 7:** Results of two-way ANOVA tests for the effects of the genotype and hippocampal subregion on the relative immunoreactivity for SREBP-1 in WT and KO mice (*n* = 5 hippocampi/group).

	WT	KO	Šidăk’s Post Hoc Test	Two-Way ANOVA
CA1	1.40 ± 0.25 ^1^	1.31 ± 0.13 ^1^	*p* = 0.8182	Genotype: F(1, 8) = 6.392, *p* = 0.0354 Subregion: F(2, 16) = 18.86, *p* < 0.0001 Interaction: F(2, 16) = 5.725, *p* = 0.0133
CA3	1.59 ± 0.07 ^1^	1.15 ± 0.25 ^1^	*p* = 0.001	
DG	1.05 ± 0.10 ^1^	1 ± 0.08 ^1^	*p* = 0.9429	

^1^ Relative optical density. The data are expressed as the means ± standard deviation. Abbreviations: ANOVA, analysis of variance; CA, *cornu ammonis*; DG, dentate gyrus; KO, SREBP-1c knockout; WT, wild-type.

## Data Availability

This paper utilized original data not used in other publications. The datasets generated and/or analyzed in the present study are available from the corresponding author upon reasonable request.
